# Soluble Calreticulin Induces Tumor Necrosis Factor-α (*TNF-α*) and Interleukin (IL)-6 Production by Macrophages through Mitogen-Activated Protein Kinase (*MAPK*) and *NFκB* Signaling Pathways

**DOI:** 10.3390/ijms15022916

**Published:** 2014-02-20

**Authors:** Cui-Cui Duo, Fang-Yuan Gong, Xiao-Yan He, Yan-Mei Li, Jun Wang, Jin-Ping Zhang, Xiao-Ming Gao

**Affiliations:** Institutes of Biology and Medical Sciences, Soochow University, 199 Ren-Ai Road, Suzhou Industrial Park, Suzhou 215123, Jiangsu, China; E-Mails: duocuicui@126.com (C.-C.D.); hexiaoyanky@163.com (X.-Y.H.); ymli@suda.edu.cn (Y.-M.L.); jwang79@suda.edu.cn (J.W.); j_pzhang@suda.edu.cn (J.-P.Z.)

**Keywords:** calreticulin, macrophage, *MAPK*, *NFκB*, scavenger receptor A

## Abstract

We have recently reported that soluble calreticulin (CRT) accumulates in the sera of patients with rheumatoid arthritis or systemic lupus erythematosus. Moreover, following self-oligomerization, soluble recombinant CRT (rCRT) polypeptides exhibit potent immunostimulatory activities including macrophage activation *in vitro* and antibody induction *in vivo*. This study was designed to further investigate the underlying molecular mechanisms for soluble CRT-induced macrophage activation. Treatment of murine macrophages with oligomerized rCRT (OrCRT) led to (i) *TNF-α* and *IL-6* transcription and protein expression without affecting intracellular mRNA stability; and (ii) IκBα degradation, *NFκB* phosphorylation and sustained *MAPK* phosphorylation in cells. Inhibition of IKK and JNK in macrophages substantially abrogated production of *TNF-α* and *IL-6* induced by OrCRT, while ERK suppression only reduced *IL-6* expression in parallel experiments. *In vitro*, fucoidan, a scavenger receptor A (SRA)-specific ligand, significantly reduced the uptake of FITC-labeled OrCRT by macrophages and subsequent *MAPK* and *NFκB* activation, thereby suggesting SRA as one of the potential cell surface receptors for soluble CRT. Together, these data indicate that soluble CRT in oligomerized form could play a pathogenic role in autoimmune diseases through induction of pro-inflammatory cytokines (e.g., *TNF-α* and *IL-6*) by macrophages via *MAPK*-*NFκB* signaling pathway.

## 1. Introduction

Calreticulin (CRT) is an endoplasmic reticulum (ER) residential glycoprotein which plays an important role in maintaining intracellular Ca^2+^ homeostasis and facilitating correct folding of major histocompatibility complex (MHC) class I molecules [1,2]. We have previously demonstrated that soluble CRT accumulates in the sera of patients with rheumatoid arthritis (RA) or systemic lupus erythematosus (SLE) [3]. Liu *et al*. reported elevated serum CRT levels in cancer patients [4]. Additionally, recombinant CRT fragments rCRT/39-272 and rCRT/18-424 are potent macrophage activators, capable of inducing tumor necrosis factor-α (*TNF-α*) and interleukin (IL)-6 production [3,5]. This may explain the correlation between serum CRT levels and autoimmune disorders, since *TNF-α* and *IL-6* represent predominant inflammatory cytokines released by activated macrophages [6,7]. Moreover, we have shown that self-oligomerization is essential for the immunostimulatory activities of rCRT [5], which is in line with previous reports that oligomerization occurs in naturally occurring CRT and CRT oligomers are more effective in peptide binding [8]. Partial unfolding conditions, such as heat shock at 42 °C and low pH (5–6) treatment, are known to favor non-covalent CRT self-oligomerization [8]. It is possible that soluble CRT in patients may self-oligomerize *in vivo*, thereby acquiring potent immunostimulatory activities and contributing to the pathogenesis in tissues. However, so far molecular mechanisms responsible for the potent immunobiological activities of soluble CRT remain elusive. This study was thus designed to investigate the signaling pathways underlying the induction of *TNF-α* and *IL-6* by oligomerized rCRT in macrophages.

## 2. Results and Discussion

### 2.1. *TNF-α* and *IL-6* Production by Murine Macrophages in Response to rCRT Stimulation

**Figure 1.**
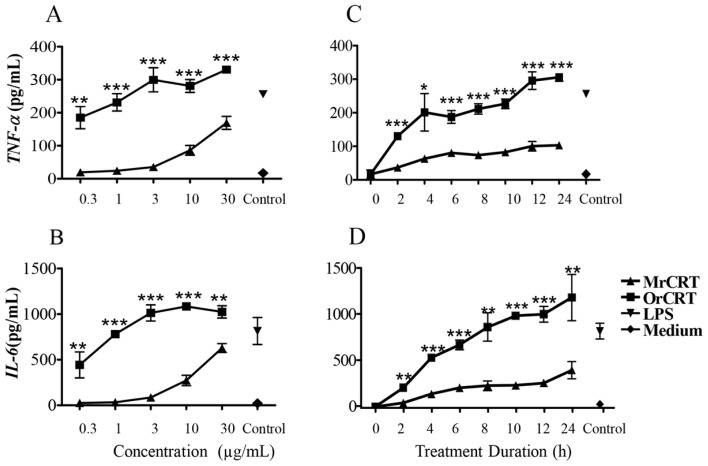
Tumor necrosis factor-α (*TNF-α*) and interleukin (IL)-6 expression in macrophages stimulated by recombinant calreticulins (rCRTs). Mouse peritoneal macrophages were stimulated with various concentrations of MrCRT or OrCRT for 24 h (**A** and **B**) or were treated with 10 μg/mL MrCRT or OrCRT for 2, 4, 6, 8, 10, 12 and 24 h (**C** and **D**). *TNF-α* (**A** and **C**) and *IL-6* (**B** and **D**) in the culture supernatant were quantitated by ELISAs. Results are expressed as mean ± SD of three independent experiments. ^*^ *p* < 0.05, ^**^ *p* < 0.01, ^***^ *p* < 0.005 oligomerized CRT (OrCRT) group *vs*. monomeric CRT (MrCRT) group.

In line with our previous report [5], oligomerized rCRT (OrCRT) dose-dependently increased the production of *TNF-α* and *IL-6* by murine peritoneal macrophages *in vitro*, while monomeric rCRT (MrCRT) was much less active (Figure 1A,B). Kinetic analysis showed that OrCRT-induced *TNF-α* and *IL-6* production by macrophages was already detectable in 2 h, reaching plateau approximately 24 h after the start of culture (Figure 1C,D). In contrast, the levels of *TNF-α* and *IL-6* remained relatively low at all time points in cultures with MrCRT.

### 2.2. *TNF-α* and *IL-6* mRNA Expression and Stability in Macrophages under rCRT Stimulation

**Figure 2.**
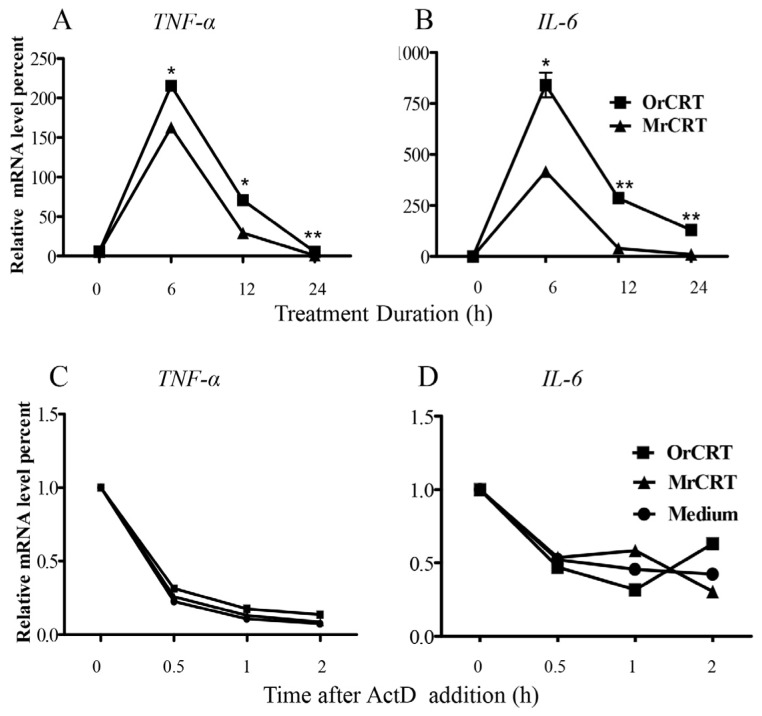
Effect of rCRTs on *TNF-α* and *IL-6* mRNA expression in macrophages. (**A**,**B**) Macrophages were stimulated by 10 μg/mL MrCRT or OrCRT for 0, 6, 12, and 24 h before harvesting for RNA isolation; (**C**,**D**) macrophages were treated with 10 μg/mL MrCRT or OrCRT for 6 h, after which Actinomycin D (5 μg/mL) was added and cells incubated further for 0, 0.5, 1, or 2 h before RNA extraction. *TNF-α* and *IL-6* mRNA levels were determined by real-time PCR. Results are expressed as mean ± SD of three independent experiments.^*^ *p* < 0.05, ^**^ *p* < 0.01 OrCRT group *vs*. MrCRT group.

The expression of short-lived inflammatory cytokines *TNF-α* and *IL-6* are known to be influenced by transcription and/or mRNA stability [9]. To investigate the effect of soluble CRT on transcription of these cytokines, we isolated total cellular RNA from peritoneal macrophages stimulated with 10 μg/mL OrCRT or MrCRT for various time periods (0–24 h) at 37 °C, then mRNA levels of *TNF-α* or *IL-6* were measured by real-time quantitative PCR. As illustrated in Figures 2A,B, the mRNA levels of *TNF-α* and *IL-6* expression induced by MrCRT or OrCRT peaked at 6 h, decreased to background level by 24 h. In accordance with protein levels (Figure 1), mRNA levels of *TNF-α* and *IL-6* in OrCRT-stimulated cells were significantly higher than that in the MrCRT-stimulated cells (Figure 2A,B). To address the effect of soluble CRT on mRNA stability, we added actinomycin D (5 μg/mL, a confirmed optimal concentration without toxicity to peritoneal macrophages) to prevent new mRNA synthesis 6 h after the culture of macrophages with rCRTs. Thereafter, cells were harvested at different time points and mRNA levels were measured by PCR to visualize the degradation of *TNF-α* and *IL-6* mRNA. As shown in Figure 2C,D, comparable degradation curves were observed in cultures with OrCRT, MrCRT or medium alone, indicating that rCRT did not affect the stability of *TNF-α* and *IL-6* mRNA in macrophages. Together, we conclude that CRT induces *TNF-α* and *IL-6* secretion by macrophages via increasing their active transcription.

### 2.3. *NFκB* Activation during rCRT-Induced *TNF-α* and *IL-6* Response

**Figure 3.**
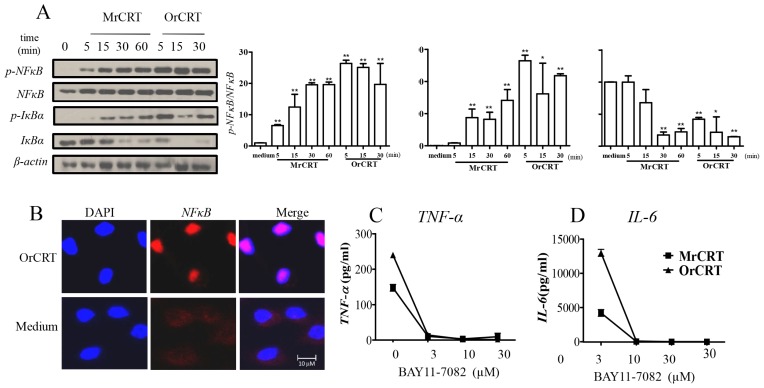
The IκB kinase (IKK)/*NFκB* pathway is required for CRT-induced *TNF-α* and *IL-6* production in macrophages. (**A**) Kinetic analysis of MrCRT or OrCRT (10 μg/mL)-induced p-IκBα and p-*NFκB* expression. Cell lysates were analyzed by SDS-PAGE and immunoblotted with antibodies against p-*NFκB*, *NFκB*, p-IκBα, IκBα. Semi-quantitative densitometric analysis of Western blots is shown in the right panels; (**B**) CRT-induced *NFκB* p65 nuclear translocation in macrophages. Peritoneal macrophages on cover-slips were treated with 10 μg/mL OrCRT for 30 min before fixation with 2% paraform. Fixed cells were then stained with rabbit anti-*NFκB* Abs and PE-conjugated donkey anti-rabbit IgG, followed by confocal laser scanning microscopy. Nuclei were visualized by DAPI; (**C**,**D**) The involvement of *NFκB* in CRT-induced *TNF-α* and *IL-6* production. Macrophages were pre-incubated with various concentrations of BAY11-7082 and then stimulated with 10 μg/mL MrCRT or OrCRT. *TNF-α* (**C**) and *IL-6* (**D**) in supernatant were determined by ELISAs. ^*^ *p* < 0.05, ^**^ *p* < 0.01 rCRT groups *vs*. Medium group.

*NFκB* is one of the common transcription factors for *TNF-α* and *IL-6* [10], and therefore likely involved in rCRT-induced macrophage activation. Dynamic analysis showed that, in macrophage cultures containing 10 μg/mL OrCRT, *NFκB* activation peaked at 5 min, considerably faster than that (15 min) with MrCRT of the same concentration (Figure 3A). As expected, OrCRT effectively induced the translocation of NF-κB from cytoplasm to nuclei of the cell (Figure 3B). Moreover, pretreatment with 3 μM BAY11-7082, an IKK-specific chemical inhibitor [11], substantially abrogated the production of *TNF-α* and *IL-6* by OrCRT-stimulated macrophages (Figure 3C,D). Clearly, *NFκB*-related pathway is involved in OrCRT-induced macrophage activation and cytokine production.

### 2.4. *MAPK* Activation during rCRT-Induced *TNF-α* and *IL-6* Response

**Figure 4.**
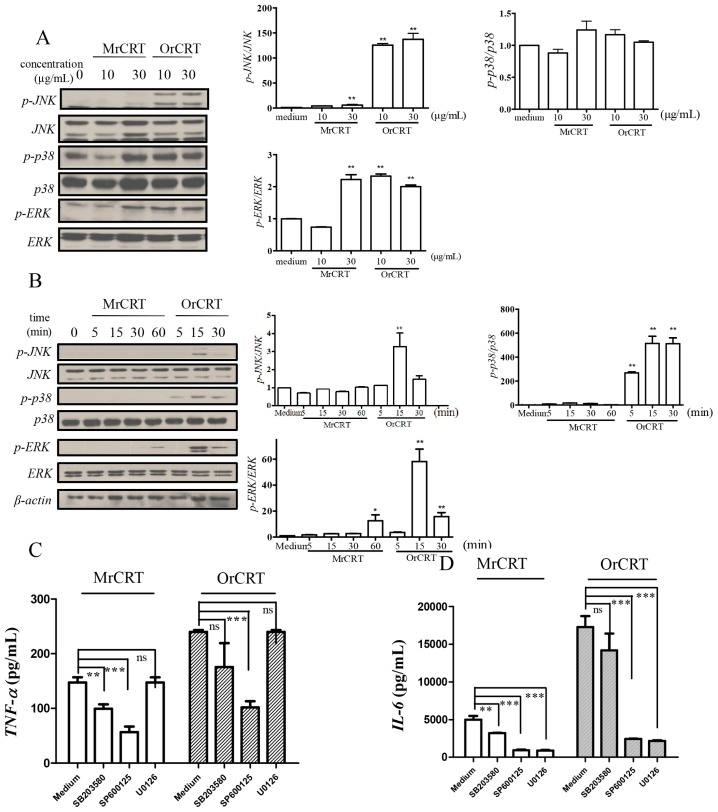
The involvement of *MAPK* in rCRT-induced *TNF-α* and *IL-6* protein expression. (**A**,**B**) *MAPK* activation in macrophages stimulated with various concentrations of MrCRT or OrCRT for 30 min (**A**), or with 10 μg/mL MrCRT or OrCRT for different time periods (**B**). Cell lysates were analyzed by SDS-PAGE and immunoblotted for total *MAPK* and phophorylated *MAPK*. Semi-quantitative densitometric analysis of Western blots was shown in the right panels; (**C**,**D**) The involvement of *MAPK* in rCRT-induced *TNF-α* and *IL-6* production. Macrophages were pretreated with 30 μM SB203580, SP600125 and U0126, and then stimulated with 10 μg/mL MrCRT or OrCRT for 24 h. *TNF-α* (**C**) and *IL-6* (**D**) in supernatant were quantitated by ELISA. ^*^ *p* < 0.05, ^**^ *p* < 0.01, ^***^ *p* < 0.005 *vs*. Medium alone group.

To determine if the mitogen-activated protein kinase (*MAPK*) signaling pathway is also involved in rCRT-induced *TNF-α* and *IL-6* expression, mouse peritoneal macrophages were incubated with MrCRT or OrCRT at various concentrations for 0–60 min. The cell lysates were subsequently assayed for the activation of *c-jun N*-terminal kinase (JNK)/stress-activated protein kinase, extracellular-signal regulated protein kinase (ERK) and p38 by Western blot. Clearly, OrCRT was more efficient than MrCRT in triggering phosphorylation of JNK, p38 and ERK (Figure 4A,B). Furthermore, SP600125, a chemical inhibitor of JNK, down-regulated *TNF-α* and *IL-6* production by mouse macrophages stimulated with rCRT (Figure 4C,D). ERK suppression by inhibiting MEK with U0126 resulted in a greater reduction of *IL-6* than *TNF-α*. In contrast, p38 inhibitor SB203580 had no significant effect on OrCRT-induced cytokine production by macrophages. Collectively, these data demonstrate that the *MAPK* signaling pathway is involved in rCRT-induced macrophage activation and cytokine production.

### 2.5. Inhibition of OrCRT Binding/Internalization by Fucoidan

**Figure 5.**
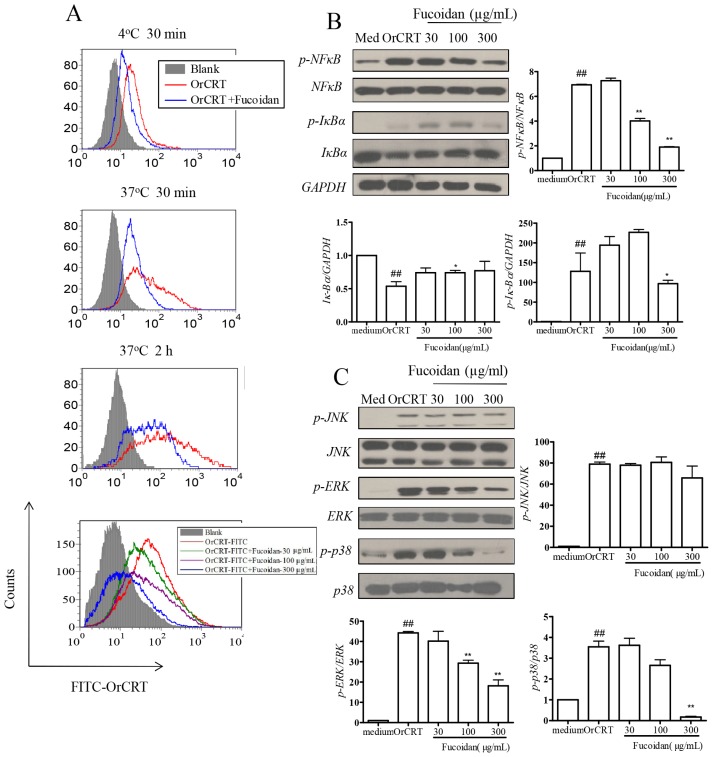
Scavenger receptor A (SRA) is involved in rCRT internalization by macrophages. (**A**) Freshly isolated mouse peritoneal macrophages were pre-incubated with fucoidan (100 μg/mL) for 2 h at 37 °C. Then FITC-OrCRT (10 μg/mL) was added and the cells were incubated further at 4 °C for 30 min, or at 37 °C for 30 min or 2 h, before flow cytometric analysis. Cells pre-treated with various concentrations of fucoidan (shown in the figure) followed by FITC-OrCRT for 30 min at 37 °C were also analyzed as control (bottom left panel). For function assay, peritoneal macrophages were pre-incubated with various concentrations of fucoidan at 37 °C for 2 h, followed by stimulation with OrCRTs (10 μg/mL) for 30 min. Cell lysates were analyzed by SDS-PAGE and immunoblotted for *NFκB* (**B**) and *MAPK* (**C**) pathway related proteins. Semi-quantitative densitometric analysis results of the Western blots are also shown. ## *p* < 0.01 OrCRT group *vs*. Medium group; ^*^ *p* < 0.05, ^**^ *p* < 0.01 vs OrCRT group.

We have previously shown that internalization of OrCRT is an important initial step in its activation of macrophages [5], but the responsible surface receptor(s) remains unknown. Interestingly, fucoidan, a specific ligand for scavenger receptor A (SRA) [12], significantly inhibited the binding and/or internalization of OrCRT-FITC to/into viable murine macrophages *in vitro* (Figure 5A–C). Furthermore, fucoidan (30–300 μg/mL) dose-dependently inhibited OrCRT-triggered *NFκB* and *MAPK* activation in macrophages (Figure 5B,C). These results indicate that SRA is a surface receptor for OrCRT and mediates, at least partially, its binding/internatilization in(to) macrophages.

### 2.6. Discussion

As one of the HSPs, soluble CRT protein exhibits potent immunological functions both *in vivo* and *in vitro*, such as activating monocytes/macrophages and expanding CD1d^hi^CD5^+^ B10 cells [13–16]. Given that elevated levels of soluble CRT in body fluids (serum and synovial fluid) correlate with autoimmune disease and cancer in humans, immunobiological activities of CRT deserves detailed investigation. In the present study, we dissected signaling pathways responsible for rCRT-induced macrophage responses. Our data revealed that CRT induces active mRNA transcription through *MAPK* and *NFκB* activation in macrophages and thereby augments their *TNF-α* and *IL-6* production. These results are supported by Basu *et al*. who reported that HSPs derived from necrotic cells could promote DC maturation through the *NFκB* pathway [17]. We also repeated these experiments using the macrophage cell line RAW264.7, very similar results were obtained (data not shown).

**Figure 6.**
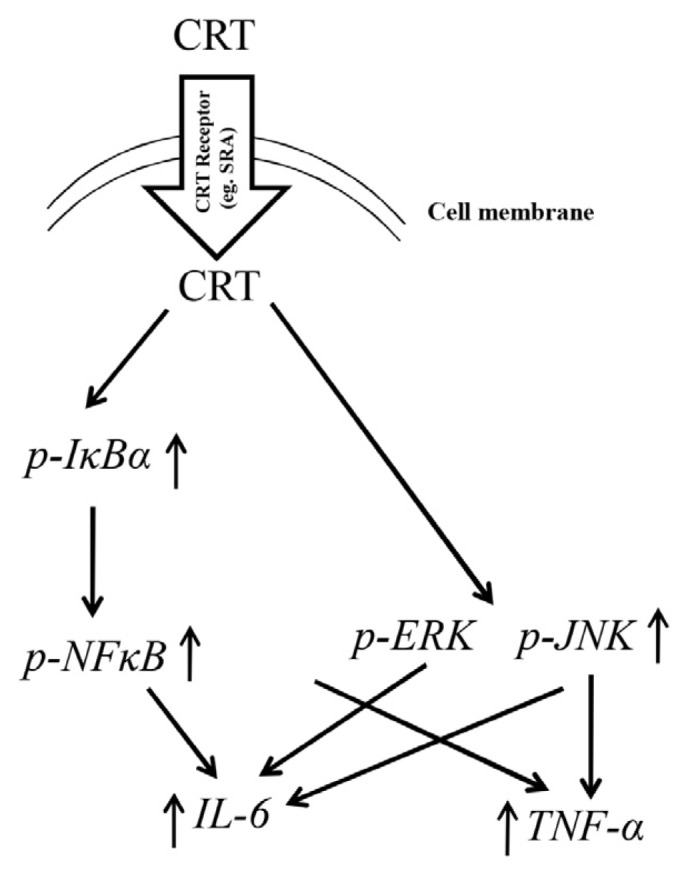
Proposed model of inflammatory cascades induced by CRT in macrophages. CRT may bind to surface receptors such as SRA on macrophages and triggers (possibily after internaliation) the activation of *IκBα-NFκB*, *p-JNK* and *p-ERK. IκBα-NFκB* and *p-JNK* mediate the production of *TNF-α* and *IL-6*, while *p-ERK* is only involved in *IL-6* expression.

CRT did not seem affect the ER stress signaling pathway (such as up-regulation of Bip), thereby sustaining the specificity of CRT’s stimulatory effect on macrophages (data not shown). CRT is known to bind common HSP receptors such as TLR2/4, LOX-1, CD36, and LRP-1 [18–25]. It has also been shown that LRP-1 serves as a macrophage surface receptor for soluble CRT [15,23]. Moreover, TLR4 and CD14 are necessary for the immune-stimulatory activity of rCRT/39-272 [3]. Here we extended these studies by showing that SRA is also a potential surface receptor for CRT. SRA, after binding to its ligand, is capable of triggering intracellular signaling through phosphorylation of phospholipase C (PLC)-γl, phosphoinositide 3 (PI3)-kinase, and protein kinase C (PKC), eventually resulting in *MAPK* activation and cytokine secretion [26]. Based upon these data, we propose the following model of soluble CRT-triggered signaling in macrophages (Figure 6): Macrophages recognize and internalize soluble rCRT oligomers via surface receptors (e.g., SRA), which subsequently trigger the activation of the IκBα/*NFκB* and *MAPK* pathways, eventually leading to the expression of *TNF-α* and *IL-6* genes. The involvement of additional receptors other than SRA in this process needs further investigation.

## 3. Experimental Section

### 3.1. Materials

Dulbecco’s modified Eagle’s medium (DMEM) and penicillin-streptomucin (100×) were obtained from Invitrogen (Calsbad, CA, USA). Fetal bovine serum was from Hyclone (Logan, UT, USA) and was heat-inactivated at 56 °C for 30 min. Antibodies against p-*NFκB*, *NFκB*, p-IκBα, IκBα, p-ERK, ERK, p-JNK, JNK, p-p38, p38 were from Cell Signaling Technology (Danvers, MA, USA) and antibodies against β-actin and GAPDH were from Santa Cruz Biotechnology (Santa Cruz, CA, USA). ECL reagents were from Thermo Pierce (Rockford, IL, USA). SP600125, U0126, SB203580, BAY11-7082 were purchased from Selleck (Houston, TX, USA). Fucoidin and carrageenan were from Sigma-Aldrich (St. Louis, MO, USA). Tauroursodeoxycholic acid was from Calbiochem (KGaA, Darmstadt). Lysosome-tracker, ER-tracker and fluo-3/AM were from Beyotime (Haimen, China). Cellular Ca^2+^ ATPase activity Kit was from Jiancheng Biotechnology (Nanjing, China). *TNF-α* and *IL-6* ELISA kit were from Biolegend (San Diego, CA, USA).

C57BL/6 female mice, 6–8 weeks of age, were from SLAC laboratory animal company (Shanghai, China). All experiments were approved by a local ethical committee.

### 3.2. Protein Purification and Separation

rCRT was purified from recombinant *E. coli* cells as previously described [5]. A Sephadex G-75 (GE Healthcare, Piscataway, NJ, USA) column of 80 × 2 cm was employed for fractionation of rCRT/18-412 oligomers and monomers.5 mL of rCRT at 10 mg/mL was loaded into the column, followed by elution with 0.9% NaCl at 20 mL/h and collected every 2 mL. The oligomers and monomers were treated by polymyxin B-agarose for three times before being used and endotoxin acitivity of oligomers and monomers were both less than 0.01 EU/μg protein.

### 3.3. Isolation of Mouse Peritoneal Macrophages

C57BL/6 mice (female, 6–8 weeks) were Intraperitoneal injection (i.p.) injected with 1.5 mL 3% thioglycollate. 72 h later, macrophages were harvested by peritoneal lavage and their purity was more than 90% as determined by FACS based on the expression of CD11b^+^/F4/80^+^.

### 3.4. Enzyme-Linked Immunosorbent Assay (ELISA) of *TNF-α* and *IL-6*

The culture media were collected and centrifuged at 12,000× *g* for 1 min. *TNF-α* and *IL-6* levels in the media were determined by ELISA as described by the manufacturer. Briefly, anti-mouse *TNF-α*/*IL-6* was used as capture antibodies and biotinylated anti-mouse *TNF-α*/*IL-6* was used as detection antibodies. Recombinant mouse *TNF-α* and *IL-6* were used as standards. The detection limit for *TNF-α* and *IL-6* is 15 ng/mL.

### 3.5. RNA Isolation and Real-Time Quantitative RT-PCR

RNA purification from cells was performed following a TRIzol/chloroform extraction. mRNA was converted into complimentary DNA (Takara, Otsu, Japan). The mRNA levels were quantified using the following primers: *TNF-α* forward primer 5′CGAGTGACAAGCCTGTAGCCC3′; *TNF-α* reverse primer 5′GTCTTTGAGATCCATGCCGTTG3′; *IL-6* forward primer 5′ACAACCACGGCCTTCCCTAC 3′; *IL-6* reverse primer TCTCATTTCCACGATTTCCCAG; β-actin forward primer 5′ATGGATGACG ATATCGCTG3′; β-actin reverse primer 5′AACACCCATTCCCTTCACAG3′. FastStart Universal SYBR Green Master mix (Roche Applied Science, Mannheim, Germany) was used as a fluorescent dye to detect the presence of double-stranded DNA. The mRNA values for each gene were normalized to internal control β-actin mRNA.

### 3.6. Western Blot Assay

Cells were washed with ice-cold PBS and subsequently lysed with RIPA lysis buffer (50 mM Tris (pH 7.4), 150 mM NaCl, 1% Triton X-100, 1% sodium deoxycholate, 0.1% SDS) with phosphatase inhibitor cocktail. Protein concentration was measured by BCA protein assay kit. Thirty microgram of total cell lysate was separated by SDS-PAGE and transferred onto PVDF membranes. Immunoblots were blocked by 5% non-fat milk in PBST buffer for 1 h at room temperature and then incubated with primary antibodies against p-*NFκB*, *NFκB*, p-IκBα, IκBα, p-ERK, ERK, p-JNK, JNK, p-p38, p38 or β-actin at 4 °C overnight. The immunoreactive bands were detected by HRP-conjugated secondary antibodies with ECL reagents.

### 3.7. Assessment of *TNF-α* and IL-6 mRNA Stability

Mouse peritoneal macrophages were treated with 10 μg/mL OrCRT or MrCRT for 6 h before the addition of actinomycin D (5 μg/mL). Total cellular RNA was extracted 0.5, 1 and 2 h after actinomycin D addition. *TNF-α* and *IL-6* mRNA levels were determined by real-time quantitative PCR. Expression levels are normalized to the mRNA amount 0 h after actinomycin D addition (set as 1).

### 3.8. Flow Cytometric Analysis

10^6^ freshly isolated peritoneal macrophages were preincubated with fucoidan (100 μg/mL) or medium for 2 h. Then cells were stained with APC-anti-F4/80 and followed by incubation with 15 μg/mL of FITC-OrCRT for 30 min at 4 °C or 37 °C. The binding and internalization of CRT by macrophages was determined by flow cytometry (BD FACS Canto II, San Jose, CA, USA).

### 3.9. Immunofluorescence Staining

Mouse peritoneal macrophages were incubated in six-well culture plates with coverslips for 2 h. Cells were further cultured with or without 10 μg/mL MrCRT or OrCRT in RPMI 1640 for 30 min. After washing twice with ice-cold PBS, cells were fixed with cold 4% paraformaldehyde at room temperature for 15 min. The staining was performed by incubating with 5% BSA/0.3% Triton X-100 in PBS at room temperature for 30 min followed by incubating with primary anti-*NFκB* p65 monoclonal antibody (1:50 dilution) overnight at 4 °C in PBS containing 1% BSA/0.3%Triton X-100, After washing with PBS, cells were incubated with PE conjugated donkey anti-rabbit IgG (10 μg/mL) for 2 h in PBS with 1% BSA/0.1%Triton X-100. Finally, the coverslips were mounted with aqueous mounting medium (with DAPI staining nuclei). The images were obtained with a fluorescence microscope (Nikon, confocal microscope system A1, Tokyo, Japan).

### 3.10. Statistical Analysis

All experiments were peformed at least 3 times and the results are expressed as mean ± standard deviation of the mean (SD). Statistical analysis was performed using the Independent-Samples *t* test or two-side paired *t* test among groups using the SPSS 14.0 program (SPSS, Chicago, IL, USA). *p* < 0.05 was considered as statistically significant.

## 4. Conclusions

In conclusion, soluble CRT oligomers are able to activate macrophages through surface receptors such as SRA and subsequently induce macrophage activation and production of *TNF-α* and *IL-6* via the *NFκB*, *MAPK* pathways. These results provide new insights on the molecular mechanisms responsible for the potential pathogenic role of soluble CRT in autoimmune diseases.
